# Fill in the blanks: developing critical appraisal skills

**DOI:** 10.1007/s40037-015-0224-6

**Published:** 2015-10-14

**Authors:** Stephen P. Hibbs

**Affiliations:** Queens Hospital, Rom Valley Way, RM7 0AG Romford, Essex UK

A great challenge in encouraging students to engage with primary literature is to help them avoid both naïve acceptance of an author’s set of conclusions as well as a superficial scatter-gun critique. An ideal learning task should require discipline in attaining comprehension of a paper before attempting to critique it. It should also encourage students to think like members of the wider scientific community rather than outsiders or critics. The latter ideal is well described by Clinchy’s ‘connected knowing’ approach in which the focus is on intimately understanding someone else’s view rather than immediately taking an adversarial role [1]. I have tried to address this challenge by asking students to ‘fill in the blanks’ in key sections of a research paper.

It is a common examination practice to test a student’s comprehension by redacting a paper’s abstract and asking the student to write their own in its place. Taking this concept further, I give a student only the methods and figures from a paper (accompanied by a short glossary) and ask them to try to decipher the authors’ aims and the conclusions from their data. We then compare their analysis with the authors’ own interpretations.

In a follow-up session, students are given the background of a different paper, and the conclusions the paper reached. Their task here is to design an appropriate experiment that could lead to data supporting the authors’ conclusions. Again, once they have completed this exercise, their suggestions are compared with the authors’ approach.

I have found this two-part exercise to compel students to read primary literature much more carefully than they would do otherwise. Furthermore, their critique of the author’s own experimental approach and conclusions is radically enhanced. A final advantage is that this approach would come more naturally to students from backgrounds where immediately criticizing someone in an authority position is not normative. I commend this as a method of developing students’ skills in critical reading and study design.

## Funding

No sources of financial support.

## Conflict of interest

No conflicts of interest or other acknowledgements.
